# Genome Sequence of *Rhodococcus* sp. Strain BCP1, a Biodegrader of Alkanes and Chlorinated Compounds

**DOI:** 10.1128/genomeA.00657-13

**Published:** 2013-10-24

**Authors:** M. Cappelletti, P. Di Gennaro, P. D’Ursi, A. Orro, A. Mezzelani, M. Landini, S. Fedi, D. Frascari, A. Presentato, D. Zannoni, L. Milanesi

**Affiliations:** Department of Pharmacy and Biotechnology (FaBiT), University of Bologna, Bologna, Italya; Department of Biotechnology and Biosciences, University of Milano-Bicocca, Milan, Italyb; CNR, Institute for Biomedical Technologies, Segrate (MI), Italyc; Department of Civil, Chemical, Environmental, and Materials Engineering, University of Bologna, Bologna, Italyd

## Abstract

*Rhodococcus* sp. strain BCP1 cometabolizes chlorinated compounds and mineralizes a broad range of alkanes, as it is highly tolerant to them. The high-quality draft genome sequence of *Rhodococcus* sp. strain BCP1, consisting of 6,231,823 bp, with a G+C content of 70.4%, 5,902 protein-coding genes, and 58 RNA genes, is presented here.

## GENOME ANNOUNCEMENT

The *Rhodococcus* genus comprises Gram-positive, nonsporulating, aerobic bacteria that are widely distributed in the environment (1). *Rhodococcus* sp. strain BCP1 (formerly *Rhodococcus aetherovorans* strain BCP1, DSM 44980) was selected from an aerobic butane-utilizing consortium as the prevailing isolate for cometabolizing chloroform, vinyl chloride, and trichloroethylene (2, 3). As BCP1 also catabolizes a wide range of aliphatic, alicyclic, and carboxylated alkanes, it represents a strain of considerable environmental and industrial interest (4).

The genome sequencing of *Rhodococcus* sp. BCP1 was performed using 454 sequencing technology (Roche GS FLX Titanium). The total numbers of the sequence reads were 668,686 from one shotgun library and 353,744 from one paired-end library (8-kb inserts). All the reads were assembled into 123 contigs using Newbler 2.6, with an N_50_ length of 237,787 bp and an average genome coverage of 65×. Based on paired-end directional information, the contigs were further ordered into 3 scaffolds, giving a total genome size of 6.2 Mb with a G+C content of 70.4%.

The annotation was performed by using the Rapid Annotations using Subsystems Technology (RAST) server (5). Two scaffolds constituting a total of ~0.2 Mb are likely to be plasmids, as they carry genetic signatures typical of *Rhodococcus* plasmids.

A total of putative 5,902 open reading frames (ORFs), 8 rRNA genes, and 50 tRNA genes were predicted. RAST annotation indicates that the strains *Rhodococcus jostii* RHA1 (score, 529) and *Rhodococcus opacus* B4 (score, 510) are the closest neighbors of strain BCP1. The subsystem categories representing the metabolisms of carbohydrates, amino acids and cofactors, vitamins, prosthetic groups, and pigments are the most abundant, as they account for 623, 565, and 429 proteins, respectively. Four hundred fifteen ORFs are involved in the metabolism of fatty acids, lipids, and isoprenoids, while 135 ORFs participate in the metabolism of aromatic compounds. Seventy-three oxygenases/hydroxylases among the 134 annotated genes are predicted to initiate the oxidation of organic compounds with industrial and environmental relevancy, such as linear alkanes, cyclic ketones, aromatic compounds (e.g., benzoate, catechol, gentisate, salicylate), aminopolycarboxylic acids, nitroalkanes, and phenylalkanoic acids. Twenty-six ORFs encode cytochrome P450 monooxygenases that catalyze regio- and stereospecific oxidation of a large number of substrates (6).

### Nucleotide sequence accession numbers.

This whole-genome shotgun project has been deposited at DDBJ/EMBL/GenBank under the accession no. AVAE00000000. The version described in this paper is version AVAE01000000.
